# Effectiveness of a Hybrid Community-Based Heart-Healthy Lifestyle Intervention: Three-Arm Randomized Controlled Trial Integrating mHealth and Motivational Interviewing

**DOI:** 10.2196/76521

**Published:** 2026-01-15

**Authors:** Jina Choo, Yura Shin, Songwhi Noh, Juneyoung Lee

**Affiliations:** 1 College of Nursing Korea University Seoul Republic of Korea; 2 Transdisciplinary Major in Learning Health Systems, Graduate School Korea University Seoul Republic of Korea; 3 Department of Biostatistics College of Medicine Korea University Seoul, Seoul Republic of Korea

**Keywords:** healthy lifestyle, cardiovascular diseases, primary prevention, mobile applications, motivational interviewing, mobile phone

## Abstract

**Background:**

Limited empirical evidence exists on the effectiveness of a hybrid approach to heart-healthy lifestyle interventions that integrates mobile health (mHealth) technology with face-to-face counseling. Moreover, its superiority over exclusive mHealth use in promoting heart-healthy behavioral outcomes within a community setting remains unclear.

**Objective:**

This study aims to evaluate the effectiveness of a hybrid community-based approach to heart-healthy lifestyle intervention incorporating a mobile app and motivational interviewing among community-dwelling adults without a history of cardiovascular disease.

**Methods:**

We conducted a 3-arm, parallel-group, randomized controlled trial with assessments at baseline and after 12 weeks. A total of 75 participants, each presenting at least 1 component of metabolic syndrome and no history of cardiovascular disease, were randomly assigned to 1 of 3 groups: hybrid (n=25), mobile (n=25), or control (n=25). Participants were recruited through an online platform. The hybrid group underwent a 12-week hybrid intervention combining a mobile app (ie, “My HeartHELP”) and face-to-face motivational interviewing led by a nursing researcher. The mobile group used only the mobile app, while the control group received written material on general heart health. The intervention was facilitated by 3 trained nursing researchers. The primary outcome was a composite score of “heart-healthy behaviors,” while secondary outcomes included scores for heart-healthy “information,” “self-efficacy,” “motivation,” and cardiovascular parameters. The trial was conducted in 2 rounds from October 2022 to May 2023. An intention-to-treat analysis was performed.

**Results:**

Of the 75 participants, 72 (96%) completed this study. Compared with the control group, both the hybrid and mobile intervention groups demonstrated significantly greater improvements in behavioral outcomes, including composite heart-healthy behavior (*F*_2,69_=7.25, *P*=.001), its theoretical predictors—heart-healthy motivation (*F*_2,69_=8.54, *P*<.001) and self-efficacy for diet (*F*_2,69_=4.87, *P*=.01) and exercise (*F*_2,69_=5.48, *P*=.006)—as well as fasting glucose levels (*F*_2,69_=3.90, *P*=.03) following the 12-week intervention. Particularly, the hybrid group—unlike the mobile group—showed significantly greater improvement in dietary behavior, a subscale of heart-healthy behavior, compared with the control group, and demonstrated significantly greater improvements in interest or enjoyment, a core subscale of intrinsic motivation, than the mobile and control groups.

**Conclusions:**

The hybrid community-based heart-healthy lifestyle intervention—integrating a mobile app and motivational interviewing—demonstrated overall effectiveness comparable to the mobile app alone, while yielding greater improvements in dietary behavior and core intrinsic motivation. These findings highlight the potential of mHealth apps as practical, stand-alone tools to promote cardiovascular health, particularly in community settings with limited access to in-person professional support. However, incorporating motivational interviewing may further enhance internalized motivation and complex behavior changes over time. Health professionals can therefore adopt mHealth either independently or in combination with motivational interviewing. Future studies should optimize integration strategies to enhance effectiveness and evaluate the long-term sustainability of such hybrid approaches.

**Trial Registration:**

ISRCTN Registry ISRCTN83643383; https://www.isrctn.com/ISRCTN83643383

**International Registered Report Identifier (IRRID):**

RR2-10.1161/circ.147.suppl_1.P147

## Introduction

Cardiovascular disease remains the leading cause of mortality worldwide [1]. To mitigate cardiovascular disease risk, the World Health Organization advocates for lifestyle modification interventions targeting smoking, poor dietary habits, physical inactivity, obesity, and alcohol consumption [2]. Evidence indicates that multiple unhealthy lifestyle behaviors significantly elevate cardiovascular disease risk [3], whereas adopting multiple healthy lifestyle behaviors can reduce all-cause mortality by up to 58%-66% [3,4]. Despite these findings, only a small proportion of individuals at risk of cardiovascular disease in the United States have achieved these recommended lifestyle changes [5]. Consequently, there is a pressing need for innovative, integrated strategies to effectively promote heart-healthy lifestyles in a comprehensive approach.

To date, mobile health (mHealth) interventions using websites, smartphones, or videoconferencing (online) have been used to improve heart health outcomes, such as cardiovascular risk parameters [6]. mHealth may help offset limited professional human resources and the time and effort required for expert-centered cardiovascular health care in the community [7]. However, evidence on the effectiveness of mHealth interventions in changing behaviors remains limited [8]. When studied, they have primarily focused on a single approach, such as physical activity [9] or weight loss [10], rather than a comprehensive strategy targeting multiple lifestyle behaviors.

Furthermore, mHealth interventions may face challenges in maximizing educational effects due to a lack of empathic support from the absence of active human interaction [11]. The primary limitation of mHealth interventions may be characterized as the lack of a robust therapeutic alliance, as highlighted by Müssener [12]. To overcome these limitations, mHealth interventions may incorporate tailored interactions using real-time feedback messages as an innovative strategy to strengthen therapeutic alliance and promote heart-healthy behavioral changes [9]. For this purpose, mHealth interventions require technologies that integrate theory-based cognitive behavioral strategies, such as goal-setting, self-monitoring, and reinforcement or feedback [13]. Based on this background, we developed a mobile app called “My HeartHELP,” incorporating 3 behavioral strategies to promote comprehensive heart-healthy behaviors: providing information, encouraging self-monitoring, and delivering real-time automatized feedback messages [14]. The behavioral strategies of “My HeartHELP” may be innovative in the ability to address multiple heart-healthy behaviors simultaneously while tailoring real-time feedback to individual behavioral outcomes. Hence, evidence on the effectiveness of the “My HeartHELP” app on heart-healthy behavioral outcomes is needed.

Nurse-led face-to-face behavioral lifestyle interventions are still required, as they elicit small-to-moderate effects on behavioral changes [15]. However, their effectiveness in modifying lifestyle behaviors remains unclear in community settings without a system that educates and supports community-dwelling individuals in self-management. Among the several behavioral intervention modes proposed, motivational interviewing has been recognized as a potential approach to facilitate behavioral changes through person-centered care, fostering active interaction between the client and provider within the client’s psychosocial context [13]. This method involves counseling techniques such as reflections, affirmations, open questions, and summarizations, which are well-received by clients because they address internal conflicts and guide goal-setting [16]. By focusing on connecting behaviors to desired outcomes and evoking an individual’s intrinsic motivation, motivational interviewing encourages people to take an active role in their own change processes [17]. In the context of behavioral interventions, motivational interviewing has proven effective in managing substance use, smoking cessation, and physical activity [18]. Therefore, motivational interviewing may complement mHealth by addressing its shortcomings and could be integrated into mHealth interventions in community settings.

A hybrid approach integrating mHealth with face-to-face motivational interviewing counseling presents a promising alternative for addressing the limitations of mHealth. This approach offers 2 key advantages. First, it combines the technological convenience of mHealth with the person-centered care of face-to-face interventions, thereby complementing the constraints of online interventions in terms of therapeutic alliance [19]. Second, in-person interactions can enhance engagement in and adherence to lifestyle behaviors recommended by health care expert providers [20]. In this regard, the American Heart Association has posited that a combined approach involving multiple modalities of behavioral interventions targeting cardiovascular risk factor reduction may be more effective than a single-modality approach [13]. Furthermore, the addition of clinical expert counseling to mobile-based cardiovascular health programs has not only been recommended [21] but has also been associated with greater reductions in systolic blood pressure when nurse-led case management was integrated with mHealth interventions, compared to mHealth alone [22]. However, empirical research on whether hybrid interventions that combine online mHealth and offline face-to-face interventions are more effective than mHealth alone remains limited.

To address the current gap in the literature, we developed and implemented a hybrid community-based approach to a heart-healthy behavioral lifestyle intervention that combines the use of the “My HeartHELP” mobile app with face-to-face motivational interviewing to maximize behavioral outcomes in cardiovascular prevention and promotion. However, little information exists on whether such a hybrid approach is effective and, furthermore, whether it is superior to mobile app use alone in achieving heart-healthy behavioral outcomes within a community setting.

Meanwhile, the Information-Motivation-Behavioral Skills (IMB) model (Multimedia Appendix 1) served as the basis for developing the behavioral strategies of the hybrid community-based approach to a heart-healthy behavioral lifestyle intervention and determining the outcome variables to evaluate its effectiveness. The IMB model explains behavioral changes using constructs such as information, motivation, and behavioral skills [23]. Theoretically, motivation in the IMB model is directly associated with behavioral skills (ie, self-efficacy) and indirectly associated with behavior via self-efficacy [23]. Motivation enhancement may effectively change heart-healthy behaviors when used as part of lifestyle interventions [24]. In particular, the hybrid community-based approach was designed to enhance personal motivation by delivering information and behavioral skills for change through mobile text messaging and individually tailored counseling, while fostering self-monitoring to promote greater self-awareness and adoption of heart-healthy behaviors. Furthermore, its group sessions—grounded in motivational interviewing—were intended to facilitate the exchange of behavioral strategies and experiences through group dynamics, thereby strengthening social support and shared norms and, in turn, enhancing social motivation.

**Figure 1:**
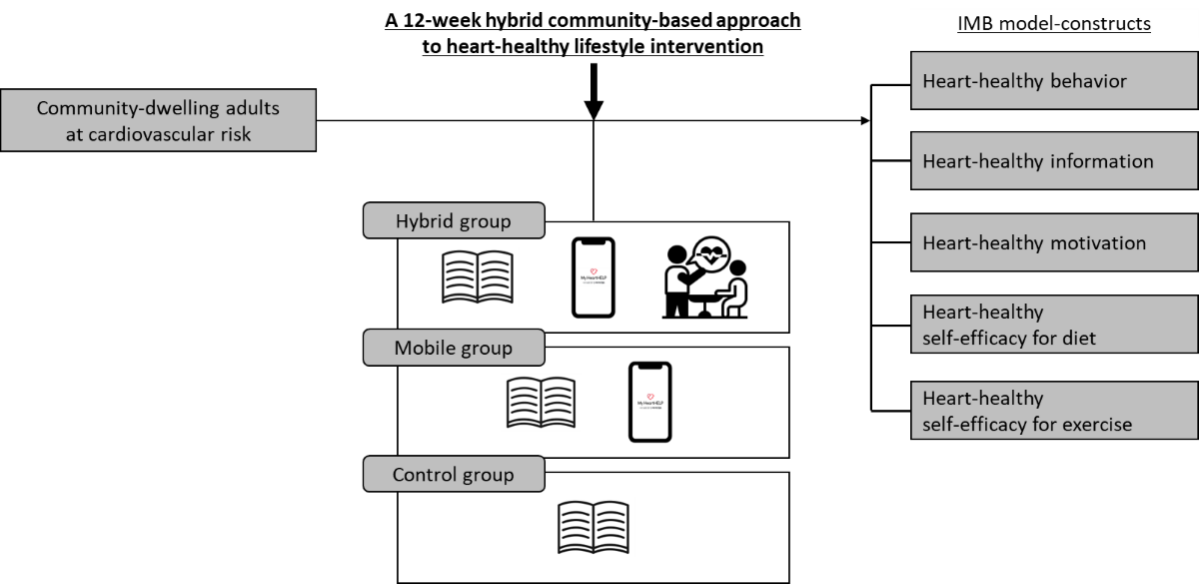
A theoretical framework of this study. The hybrid group received a 12-week hybrid intervention combining a mobile app (ie, “My HeartHELP”) and face-to-face motivational interviewing; the mobile group used the mobile app (My HeartHELP), and the control group received a brochure on general heart-health. IMB: Information-Motivation-Behavior Skills.

Therefore, the hybrid intervention based on the IMB model has a theoretical foundation aimed at informing individuals about cardiovascular health through frequent text messaging (heart-healthy information) and encouraging them through mHealth and motivational interviewing to promote intrinsic motivation (heart-healthy motivation). This, in turn, enhances behavioral skills (heart-healthy self-efficacy for diet and exercise) through cognitive-behavioral strategies and facilitates behavioral changes (heart-healthy behavior; Figure 1).

We aimed to examine the effectiveness of a 12-week hybrid community-based heart-healthy lifestyle intervention among community-dwelling adults without a history of cardiovascular disease (Figure 1). We tested 2 hypotheses: first, a mobile group would be more likely to improve heart-healthy information, motivation, behavioral skills, and behavior (ie, IMB model-constructs) and cardiovascular parameters than a control group. Second, a hybrid group would be more likely to improve these outcomes than the mobile or control groups.

## Methods

### Study Design

This study (clinical trial number ISRCTN83643383) was a randomized controlled trial with 3 arms, parallel groups, and a 12-week follow-up period: a hybrid group, a mobile app group, and a control group. The pretest (T1) was conducted before the 12-week intervention, while the posttest (T2) was conducted after the intervention. This study was conducted in 2 cohort rounds to facilitate the effective implementation of the intervention by optimizing resources within a small research team. The rationale for the separate rounds was based on the study by Burke et al [25]. The first round was conducted between November 2022 and February 2023, and the second round between February and May 2023 in a community-based setting in Seoul, South Korea.

As this study adopted a prospective, randomized, open-label, blinded end point design [26], double blinding was not feasible due to the nature of the interventions, which involved mobile app use and counseling. Specifically, neither study participants nor interviewers were blinded to this study’s groups, but outcome assessors were blinded. We report our trial using the CONSORT (Consolidated Standards of Reporting Trials) guidelines (checklist provided in Multimedia Appendix 2) [27].

### Participants

This study’s participants comprised 75 community-dwelling adults, and the inclusion criteria were as follows: (1) aged between 20 and 64 years, and (2) having at least one metabolic syndrome according to the National Cholesterol Education Program Expert Panel [28]. The exclusion criteria were as follows: (1) medically diagnosed with diabetes mellitus and taking hypoglycemics or insulin, (2) medically diagnosed with either cardiovascular or psychiatric disease (ie, major depression or anxiety disorder), (3) physical activity limitations, (4) cognitive problems, and (5) inability to use mobile apps.

Participants were recruited using a notice on the internal bulletin board of the university with which the researchers were affiliated, as well as on the external bulletin boards of mobile communication platforms. The first round of recruitment was conducted from October 17 to November 11, 2022, and the second round from January 11 to February 3, 2023. When interested candidates contacted the researchers by phone to participate in this study, the researchers determined whether they were eligible and informed them that they could be randomly assigned to 3 groups. After agreeing to participate, participants were asked to attend a preliminary investigation session in person, where the purpose, methods, and procedures of this study were explained in detail based on this study’s protocol, and written informed consent was obtained.

### Sample Size

The minimum sample size per group was calculated based on the effect size of the composite heart-healthy behavior—the primary outcome variable in this study—between the mobile and control groups. According to Park [29], the mean difference (MD; ∆=change in mean composite heart-healthy behavior score from pretest to posttest) was 0.66 and –0.15 in intervention and control groups, respectively. However, we conservatively assumed that there would be no difference in the composite heart-healthy behavior between the pre- and posttests (ie, 0.0) in the control group. Thus, the effect size assumed in this study was 0.66.

The SD of the mean score changes for each group was estimated as follows, based on the results from the dissertation by Park [29]: pretest score SDs were assumed to be 0.47 and 0.56, and the posttest score SDs were 0.41 and 0.50 for the intervention and control groups, respectively. By conservatively assuming a correlation coefficient of 0.5 between pre- and posttests, and using the method proposed by Abrams et al [30], we projected the SD of the mean score changes from pre- to posttests to be 0.443 and 0.533 for the intervention and control groups, respectively. With these SDs of mean score changes and, under a 2-sided significance level (α) of 2.5%, a minimum of 20 subjects per group was needed to ensure a statistical power (1-β) of 95% to detect the effect size of 0.66. Assuming a 20% dropout rate during this study’s period, we planned to enroll 25 participants in each group. Statistical software PASS 2020 (version. 20.0.8; NCSS LLC) was used to calculate the sample size.

### Random Allocation

Random allocation in this study was conducted by the principal investigator using an age- and gender-stratified block randomization method with an allocation ratio of 1:1:1 across the 3 study arms. A randomization list was generated for each of the 10 age- and gender-stratified groups (ie, 20-29, 30-39, 40-49, 50-59, and 60-65 years, by gender) using R software (version 3.6.0; R Foundation for Statistical Computing). Study participants were listed in the order in which they were recruited, and each participant was assigned to an appropriately stratified group based on age and sex. Finally, a total of 75 participants were allocated to the hybrid (n=25), mobile (n=25), or control (n=25) groups.

### Study Intervention

**Figure 2:**
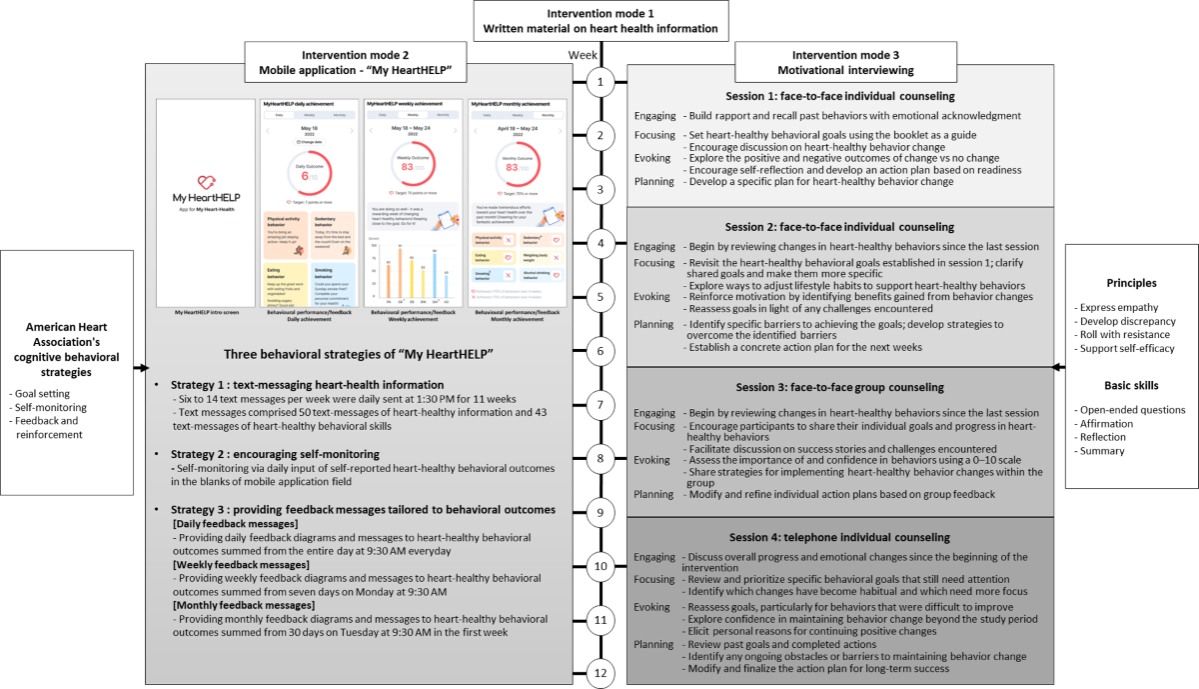
An outline for the 12-week hybrid community-based heart-healthy lifestyle intervention. The 12-week hybrid intervention consisted of the three intervention modes: (1) intervention mode 1: written materials on general heart-health information, (2) intervention mode 2: the “My HeartHELP” mobile app, and (3) intervention mode 3: motivational interviewing counseling. General heart health information was delivered via a brochure, including information on cardiovascular diseases, cardiovascular risk factors, and general heart-healthy lifestyle changes. Please see Multimedia Appendix 3 for a larger version of this figure.

An outline and details of the hybrid intervention are presented in Figure 2, which contains three intervention modes: (1) intervention mode 1: written materials on general heart-health information, (2) intervention mode 2: the “My HeartHELP” mobile app [14], and (3) intervention mode 3: motivational interviewing counseling. General heart health information was delivered via a brochure, including information on cardiovascular diseases, cardiovascular risk factors, and general heart-healthy lifestyle changes. The duration of intervention modes 2 and 3 was 12 weeks. Specifically, the mobile app was designed for daily use throughout the intervention period, while motivational interviewing was delivered in 4 sessions, each lasting 2 hours, conducted at weeks 1, 4, 8, and 10.

The hybrid group participated in all 3 intervention modes. The mobile group received 2 intervention modes of written materials and the “My HeartHELP” app. The control group received only the written materials.

Participants in both the hybrid and mobile groups attended separate 1-hour preintervention education sessions, which provided an overview of the 12-week intervention schedule and instructions on using the “My HeartHELP” app. A printed booklet containing a QR code for app download was distributed, and participants were informed that the app was freely available for research use. Each session also included a hands-on practice session, during which the research team assisted participants with app registration and guided them in entering sample data into the app.

The “My HeartHELP” app was implemented as an online mode, consisting of 3 strategies: strategy 1—text messaging with heart-health information, strategy 2—encouraging the self-monitoring of 6 lifestyle behaviors (physical activity, nonsedentary behavior, healthy dietary behaviors, nonsmoking, nonalcohol binge drinking, and daily weighing of body weight) [14], and strategy 3—providing automated or tailored feedback messages tailored to individual behavioral outcomes from self-monitoring (Figure 2). The text messaging intervention was delivered daily at 1:30 PM over a period of 1 to 11 weeks and comprised 50 messages with heart-health information and 43 messages focused on heart-healthy behavioral skills. Encouraging self-monitoring was also a daily practice, requiring participants to record behavioral outcomes for the 6 behaviors by entering data into the designated input fields of the app. Automated or tailored feedback messaging, including diagrams and texts, was daily, weekly, and monthly based. As a preparatory step toward developing automated and tailored feedback messaging, the research team established behavioral target goals and their ranges for daily, weekly, and monthly outcomes based on 6 heart-healthy behaviors. According to the ranges, a pool of feedback text messages was developed and embedded into the app’s algorithm. For daily outcomes, scores obtained from self-monitoring of the 6 heart-healthy behaviors during the previous day were categorized as either >7 or <7, with the daily goal set at >7 out of a possible 10 points. The daily feedback messages were presented as a diagram and texts at 9:30 AM every day. For weekly outcomes, the average scores over the previous 7 consecutive days were converted to a 100-point scale and classified into 3 categories: ≥70, 50-69, and <50. The weekly goal was set at ≥70. The weekly feedback messages were delivered on Monday at 9:30 AM. For monthly outcomes, the mean scores across the preceding 4 weeks were dichotomized into ≥70 and <70. The monthly feedback messages were delivered as diagrams and text messages on Tuesday at 9:30 AM in the first week. The app automatically delivered tailored feedback messages through an embedded algorithm that operated according to predefined behavioral target goals and their corresponding ranges. Further details are available in the feasibility and acceptability study of the “My HeartHELP” mobile app [14].

Motivational interviewing counseling was an offline face-to-face intervention conducted in 4 sessions: sessions 1 (week 1) and 2 (week 4) for individualized counseling, session 3 (week 8) for group counseling, and session 4 (week 10) for telephone counseling (Figure 2). Each session followed the core processes of engaging, focusing, evoking, and planning, as outlined in Figure 2 [17]. Engaging focused on building trust, understanding participants’ unique perspectives, reviewing past behaviors, and evaluating emotional and behavioral changes [17]. Focusing aimed to clarify shared goals, making them more specific and actionable, while evoking strengthened participants’ motivations for change by imagining potential outcomes and assessing their importance and confidence. Planning facilitated the development of practical strategies by identifying barriers to behavior change and determining ways to overcome them. The intervention was tailored to individual lifestyle contexts and used the principles of facilitating empathy, discrepancy, resistance, and self-efficacy along with open-ended questions, affirmations, reflections, and summaries techniques. Additional details are presented in Figure 2.

### Fidelity of Intervention

To ensure the fidelity of the “My HeartHELP” app implementation, 2 measures used in previous studies [14,31] were applied: access and self-monitoring rates. Participants in both the hybrid and mobile groups were instructed to access the app daily and self-monitor their behavioral outcomes (ie, fill in the blanks of the behavioral components) daily for 12 weeks. Among those enrolled in the hybrid and mobile groups, 36/47 (76.6%) accessed the app at least once daily, while 45.7/47 (97.3%) engaged daily in self-monitoring of heart-healthy behaviors throughout the 12 weeks.

To ensure the fidelity of motivational interviewing, all authors completed training and certification through motivational interviewing courses, including 10 hours of fundamental information and 8 hours focused on health care [32]. One author implemented the motivational interviewing sessions over 12 weeks as an interventionist, with assistance from another author. To maintain counseling quality, an activity sheet template was developed and used for each session. Regarding attendance adherence, participants in the hybrid group attended 23/23 (100%) of the sessions.

### Measures

#### Heart-Healthy Behavioral Outcomes Variables

The primary outcome was the composite score of heart-healthy behaviors. Secondary outcomes included scores for heart-healthy knowledge, heart-healthy self-efficacy, heart-healthy motivation, and cardiovascular parameters. All outcomes were measured at baseline (T1) and after 12 weeks (T2) using self-reported questionnaires, anthropometric measurements, and blood sampling.

Composite heart-healthy behavior refers to the extent to which participants practiced heart-healthy behaviors for cardiovascular health. This was measured using the Management Behaviors of Metabolic Syndrome Evaluation Tool developed by Kang [33] for individuals with metabolic syndrome. This tool consists of 36 questions: 8 on “physical activity and weight control,” 16 on “dietary habits,” 3 on “drinking and smoking,” 3 on “stress,” 2 on “sleep and rest,” and 4 on “health check-up and management.” The average score was calculated by summing the scores for each item (rated from “never” to “always” on a 4-point Likert scale) and dividing it by the number of items. Higher scores indicated greater heart-healthy behavior. Cronbach α was 0.92 in the study by Kang [33] and 0.91 in this current study.

*Heart-healthy information* refers to the level of knowledge regarding cardiovascular disease prevention. This was measured using the Heart-Healthy Information Questionnaire developed by Choo et al [34]. This tool consists of 50 questions, each answered as “true,” “false,” or “don’t know.” Participants who answered correctly received 1 point. If participants answered incorrectly or did not know, they received 0 points. The total score ranged from 0 to 50 points. The Kuder-Richardson formula 20 was 0.85 in the study by Choo et al [34] and 0.81 in this current study.

*Motivation* refers to intrinsic motivation levels for practicing heart-healthy behaviors. It was measured using the Intrinsic Motivation Inventory developed by McAuley et al [35], which we modified for the present study. Based on the inventory guidance indicating that subscales may be selectively used according to the study’s focus [36], we used 5 of the 7 subscales, excluding 2 subscales. The pressure or tension subscale was excluded because it is more relevant to stressful activities such as competitive sports [37,38] than to heart-healthy behaviors. The relatedness subscale was also excluded because its items capture relational closeness—such as perceived distance, trust, and friendship with specific individuals—which are less relevant to the autonomous, self-directed process of adopting heart-healthy lifestyle habits. Consequently, 5 of the original 7 subscales were used—interest or enjoyment (7 items), perceived competence (6 items), effort or importance (5 items), perceived choice (7 items), and value or usefulness (7 items)—yielding a total of 32 items. The English version of the modified Intrinsic Motivation Inventory was minimally adjusted to fit heart-healthy behaviors, then translated into Korean separately by 3 Korean nursing scholars and consolidated into a single Korean version. A native English speaker back-translated this version, and the final English version was reviewed and conﬁrmed by the original translators. Participants rated items using a 7-point Likert scale ranging from “completely disagree” to “strongly agree,” with responses summed and averaged for each participant. Cronbach α was 0.85 in the study by McAuley et al [35] and 0.88 in this current study.

*Heart-healthy self-efficacy* refers to participants’ confidence in their ability to engage in heart-healthy eating and exercise. It was measured using 2 instruments: the Self-Efficacy for Diet and Self-Efficacy for Exercise tools developed by Sallis et al [39]. The Self-Efficacy for Diet instrument comprises 20 items, while the Self-Efficacy for Exercise instrument has 12 items. Scores for eating and exercise habits were calculated by summing the responses on a 5-point Likert scale, ranging from “I cannot do this at all” to “I can definitely do this.” Higher scores indicate higher self-efficacy levels. Cronbach α for eating was 0.84 in the study by Shin and Lach [40] and 0.91 in this current study. Cronbach α for exercise was 0.90 in the study by Sallis et al [39] and 0.93 in this current study.

#### Cardiovascular Parameters

*Cardiovascular parameters* comprised BMI, waist circumference, blood pressure, fasting glucose, total cholesterol, low-density lipoprotein cholesterol, and high-density lipoprotein cholesterol. Body weight was measured after an overnight fast using the InBody270 scale (InBody Co, Ltd). Before weighing, participants wore a study-provided gown and removed their shoes. Height was measured using a wall-mounted stadiometer. BMI was calculated as weight (kg) divided by height (m^2^). Waist circumference (cm) was measured twice using a Gullick II measuring tape at the midpoint between the lowest rib and the iliac crest, with the average of the 2 measurements used. Blood samples were drawn from the antecubital vein in the morning after a 10-hour overnight fast, without participants taking any current medications, including antihypertensive or lipid-lowering drugs. The samples were analyzed as described in a previous study [41].

### Statistical Analysis

Participants’ demographic and baseline characteristics were summarized using descriptive statistics, including frequency, percentage, mean, and SD. To test the homogeneity of the 3 groups’ baseline measures, chi-square tests were used for categorical variables, while a 1-way ANOVA was performed for continuous variables (ie, age and study variables).

Both intention-to-treat and per-protocol approaches were used. The intention-to-treat analysis included all randomized participants regardless of adherence to the intervention, whereas the per-protocol population consisted of those who strictly adhered to the intervention protocol [42]. Missing values, which occurred due to nonresponse to the measurements, were not imputed but analyzed as observed. Analysis of covariance was used to compare outcome variables among the hybrid, mobile, and control groups at T2. In this analysis, T2 scores served as the dependent variable, study groups as the main exposure variables, and baseline scores for each respective outcome variable as confounders to adjust for individual differences at the start of the intervention. As a post hoc analysis, the Tukey least significant difference multiple comparison test was performed to identify significant differences between study groups in outcome variable changes. The analyses further examined significant group differences in the subscales of composite heart-healthy behavior and motivation. All statistical analyses were conducted using SPSS/WIN (version 28.0; SPSS Inc), with a 2-sided *P*<.05 considered statistically significant.

### Ethical Considerations

This study was approved by the Institutional Review Board of Korea University (KUIRB-2022-0287-01). All participants provided written informed consent. All procedures were performed in accordance with the ethical standards of the Institutional Research Committee and the 2013 Declaration of Helsinki [43].

Safety and security procedures related to the use of the mobile app were carefully implemented. To ensure data privacy, access to the secure data server was restricted to a single designated member of the research team. Data were downloaded and processed offline to enhance data protection and confidentiality. Preintervention education sessions were conducted to inform participants about relevant safety and privacy measures. In addition, real-time user support was offered via KakaoTalk (Kakao Corp), a widely used mobile messaging platform in South Korea, enabling participants to directly communicate with the research team throughout the intervention period. Participants in the control group, mobile group, and hybrid group who completed only the pretest received a KR ₩30,000 (US $21) gift voucher. Participants who successfully completed all stages of the study received monetary compensation according to group assignment: KR ₩150,000 (US $104) for the control group, KR ₩250,000 (US $172) for the mobile group, and KR ₩300,000 (US $207) for the hybrid group. All details related to compensation were fully disclosed to participants during the informed consent process before their enrollment in this study.

## Results

### Participants’ General Characteristics

**Figure 3:**
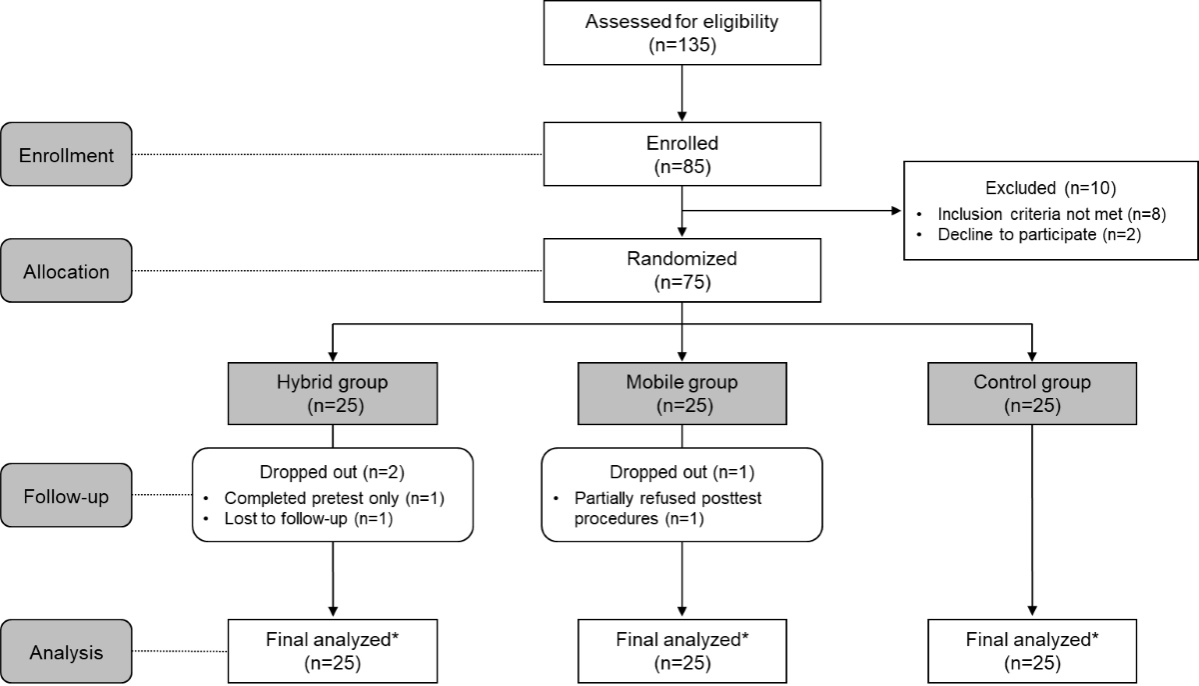
Participant flow in this study. The hybrid group received a 12-week hybrid intervention combining a mobile app (ie, “My HeartHELP”) and face-to-face motivational interviewing; the mobile group used the mobile app, and the control group received written material on general heart-health information. *All participants were included in an intention-to-treat analysis.

Three participants dropped out of this study for the following reasons: one participant from the hybrid group due to time constraints (n=1), another from the hybrid group due to loss to follow-up (n=1), and one from the mobile group who declined to participate in the posttest (n=1; Figure 3). Since the findings from both the intention-to-treat and per-protocol analyses yielded identical statistical significance, the results are presented based on the intention-to-treat analysis.

The participants had a mean age of 43.6 (SD 11.3) years (Table 1). Of all participants, 46 out of 75 (61.3%) were women, and 64 out of 75 (85.3%) were college-educated. Additionally, 32 out of 75 (42.7%) had a median income level greater than or equal to that of the general South Korean population [44]. Among the participants, 52 out of 75 (69.3%) were used, and 16 out of 75 (21.3%) were taking antihypertensive or lipid-lowering medications. No significant differences in general characteristics were observed between the groups (Table 1).

**Table 1:** Participants’ general characteristics (N=75) those of the general South Korean population [44]. Among the participants, 52/75 (69.3%) were employed, and 16/75 (21.3%) were taking antihypertensive or lipid-lowering medications. No significant differences in general characteristics were observed between the groups.

			All (N=75)		Hybrid group (n=25)		Mobile group (n=25)		Control group (n=25)		Chi-square (*df*)		*F* test (*df*)		*P* value
Age (years), mean (SD)			43.6 (11.3)		43.4 (11.0)		43.9 (12.3)		43.6 (11.0)				0.01 (2, 69)		.99
**Gender,** **n (%)**											0.11 (2, 69)				.94
	Women	46 (61.3)		16 (64)		15 (60)		15 (60)							
	Men	29 (38.7)		9 (36)		10 (40)		10 (40)							
**Education,** **n (%)**											0.21 (2, 69)				.90
	More than college-educated	64 (85.3)		22 (88)		21 (84)		21 (84)							
	Less than college-educated	11 (14.7)		3 (12)		4 (16)		4 (16)							
**Monthly household income^a^, 10,000 won, n (%)**											2.07 (2, 69)				.36
	>500	32 (42.7)		11 (44)		8 (32)		13 (52)							
	<500	43 (57.3)		14 (56)		17 (68)		12 (48)							
**Employed,** **n (%)**											1.63 (2, 69)				.44
	Yes	52 (69.3)		15 (60)		18 (72)		19 (76)							
	No	23 (30.7)		10 (40)		7 (28)		6 (24)							
**Medications^b^, n (%)**											1.11 (2, 69)				.57
	Yes	16 (21.3)		5 (20)		4 (16)		7 (28)							
	No	59 (78.7)		20 (80)		21 (84)		18 (72)							

^a^Monthly household income was classified using a cutoff of 5,000,000 won (US $3450; IQR 1767-5300), which represents the median income of the general population in South Korea [44].

^b^Taking either antihypertensives or lowering lipids medications.

### Heart-Healthy Behavioral Outcome Variables

Table 2 summarizes the results of the differential effects of the 3 groups on the primary and secondary outcome variables over a 12-week intervention period. Significant differences were observed among the 3 groups in the scores for composite heart-healthy behavior (*F*_2,69_=7.25, *P*=.001), motivation (*F*_2,69_=8.54, *P*<.001), self-efficacy for diet (*F*_2,69_=4.87, *P*=.01), and self-efficacy for exercise (*F*_2,69_=5.48, *P*=.006). Compared to the control group, the hybrid and mobile groups demonstrated significantly greater increases in the scores for composite heart-healthy behavior (hybrid vs control: MD 0.37, SD 0.10, *P*<.001; mobile vs control: MD 0.26, SD 0.10; *P*=.01), motivation (hybrid vs control: MD 0.74, SD 0.18; *P*<.001; mobile vs control: MD 0.42*,* SD 0.18; *P*=.02), self-efficacy for diet (hybrid vs control: MD 8.44, SD 2.83; *P*=.004; mobile vs control: MD 6.29, SD 2.77; *P*=.03), and self-efficacy for exercise (hybrid vs control: MD 5.01, SD 2.27; *P*=.03>; mobile vs control: MD 7.23, SD 2.24; *P*=.002). There were no significant differences in the heart-healthy information scores among the 3 groups.

**Table 2:** Effectiveness of a hybrid community-based heart-healthy lifestyle intervention on heart-healthy behavioral outcome variables (N=75). T1 and T2 refer to the baseline time point and the time point after 12 weeks, respectively.

					Hybrid group (n=25)		Mobile group (n=25)		Control group (n=25)		*F* test (*df*)			*P* value	
**Composite heart-healthy behavior, mean (SD)**															
	**Overall**											7.25 (2, 69)			.001
		T1		2.4 (0.46)		2.4 (0.47)		2.4 (0.42)							
		T2		2.9 (0.46)^a^		2.8 (0.42)^a^		2.5 (0.50)^b^							
	**Physical activity and weight control**											4.02 (2, 69)			.02
		T1		1.9 (0.73)		2.2 (0.71)		2.0 (0.57)							
		T2		2.5 (0.56)^a^		2.7 (0.75)^a^		2.2 (0.68)^b^							
	**Dietary habits**											5.04 (2, 69)			.009
		T1		2.3 (0.50)		2.3 (0.60)		2.3 (0.55)							
		T2		2.9 (0.51)^a^		2.7 (0.58)^a,b^		2.5 (0.62)^b^							
	**Drinking and smoking**											0.06 (2, 69)			.94
		T1		3.3 (0.91)		3.2 (0.81)		3.1 (0.92)							
		T2		3.3 (0.85)		3.3 (0.91)		3.2 (0.89)							
	**Stress**											1.54 (2, 69)			.22
		T1		3.2 (0.69)		3.0 (0.70)		2.9 (0.53)							
		T2		3.4 (0.54)		3.2 (0.66)		3.0 (0.63)							
	**Sleep and rest**											1.88 (2, 69)			.16
		T1		2.9 (0.69)		2.7 (0.64)		2.8 (0.60)							
		T2		3.1 (0.73)		3.1 (0.74)		2.8 (0.74)							
	**Health management**											8.67 (2, 69)			<.001
		T1		2.4 (0.59)		2.2 (0.55)		2.3 (0.69)							
		T2		2.9 (0.59)^a^		2.7 (0.60)^a^		2.3 (0.70)^b^							
**Heart-healthy information, mean (SD)**												2.68 (2, 69)			.08
			T1		43.0 (4.04)		41.9 (4.04)		38.6 (6.49)						
			T2		45.5 (3.03)		44.0 (3.01)		41.6 (4.12)						
**Heart-healthy motivation, mean (SD)**															
	**Overall**											8.54 (2, 69)			<.001
		T1		4.4 (0.80)		4.6 (0.74)		4.4 (0.56)							
		T2		5.5 (0.60)^a^		5.2 (0.80)^a^		4.7 (0.76)^b^							
	**Effort or importance**											9.45 (2, 69)			<.001
		T1		3.8 (1.22)		4.3 (1.24)		3.9 (1.07)							
		T2		5.5 (0.73)^a^		5.4 (0.93)^a^		4.5 (1.15)^b^							
	**Interest or enjoyment**											10.48 (2, 69)			<.001
		T1		3.7 (1.24)		4.1 (1.21)		4.0 (0.95)							
		T2		5.5 (1.02)^a^		5.1 (1.09)^b^		4.4 (1.09)^c^							
	**Perceived choice**											1.83 (2, 69)			.17
		T1		4.7 (0.70)		4.6 (0.61)		4.5 (0.78)							
		T2		5.0 (0.65)		4.9 (0.60)		4.6 (0.87)							
	**Perceived competence**											3.28 (2, 69)			.04
		T1		3.5 (1.28)		3.9 (1.13)		3.8 (0.83)							
		T2		4.8 (1.04)^a^		4.7 (1.16)^a,b^		4.2 (1.13)^b^							
	**Value or usefulness**											1.85 (2, 69)			.16
		T1		5.9 (0.95)		6.0 (0.80)		5.7 (0.81)							
		T2		6.5 (0.67)		6.1 (1.21)		5.9 (0.95)							
**Heart-healthy self-efficacy for diet, mean (SD)**												4.87 (2, 69)			.01
			T1		71.5 (13.94)		71.2 (13.79)		75.4 (11.86)						
			T2		79.8 (11.48)^a^		77.3 (10.62)^a^		73.1 (12.89)^b^						
**Heart-healthy self-efficacy for exercise, mean (SD)**												5.48 (2, 69)			.006
			T1		37.4 (11.26)		38.9 (10.25)		36.2 (8.32)						
			T2		40.3 (10.07)^a^		43.5 (7.43)^a^		34.8 (10.32)^b^						

^a^Different superscripts indicate a statistically significant difference by Tukey least significant difference multiple comparison.

^b^Different superscripts indicate a statistically significant difference by Tukey least significant difference multiple comparison.

^c^Different superscripts indicate a statistically significant difference by Tukey least significant difference multiple comparison.

Regarding the subscales of heart-healthy behavior, significant group differences were observed in physical activity and weight control (*F*_2,69_=4.02, *P*=.02), dietary habits (*F*_2,69_=5.04, *P*=.009), and health management (*F*_2,69_=8.67, *P*<.001; Table 2). Specifically, both the hybrid and mobile groups showed significantly greater increases in physical activity and weight control (hybrid vs control: MD 0.38, SD 0.17; *P*=.03; mobile vs control: MD 0.45, SD 0.17; *P*=.01), as well as health management (hybrid vs control: MD 0.62, SD 0.16; *P*<.001; mobile vs control: MD 0.49, SD 0.16; *P*=.002), compared with the control group. Moreover, the hybrid group (MD 0.42, SD 0.13; *P*=.002), but not the mobile group, demonstrated significantly greater improvements in dietary habit scores compared with the control group.

Regarding the subscales of heart-healthy motivation, significant group differences were observed in effort or importance (*F*_2,69_=9.45, *P*<.001), interest or enjoyment (*F*_2,69_=10.48, *P*<.001), and perceived competence (*F*_2,69_=3.28, *P*=.04; Table 2). The hybrid group demonstrated significantly greater increases in interest or enjoyment than both the mobile (MD 0.54, SD 0.27; *P*=.045) and control groups (MD 1.21, SD 0.27; *P*<.001), while the mobile group also showed a significantly greater increase than the control group (MD 0.67, SD 0.26; *P*=.01). Both the hybrid (MD 1.00, SD 0.25; *P*<.001) and mobile (MD 0.79, SD 0.24; *P*=.002) groups showed significantly greater increases in effort or importance compared to the control group; however, there were no significant differences between the hybrid and mobile groups. Finally, the hybrid group (MD 0.70, SD 0.28; *P*=.01), but not the mobile group, demonstrated a significantly greater increase in perceived competence compared to the control group.

### Cardiovascular Parameters

Table 3 summarizes the differential effects of the 3 groups on cardiovascular parameters over 12 weeks. Significant differences were observed between the 3 groups in fasting glucose levels (*F*_2,69_=3.90, *P*=.03), with significant differences between the hybrid and control groups (*P*=.02) and the mobile and control groups (*P*=.02). However, there were no significant differences in the other parameters among the 3 groups.

**Table 3:** Effectiveness of a hybrid community-based heart-healthy lifestyle intervention on cardiovascular parameters (N=75). T1 and T2 refer to the baseline time point and the time point after 12 weeks, respectively.

		Hybrid group (n=25)	Mobile group (n=25)	Control group (n=25)	*F* test (*df*)		*P* value	
**BMI (kg/m^2^), mean (SD)**						0.82 (2, 69)		.45
	T1	28.2 (4.84)	26.6 (3.73)	26.2 (4.14)				
	T2	27.9 (4.79)	26.3 (3.93)	26.2 (4.01)				
**WC^a^ (cm), mean (SD)**						0.20 (2, 69)		.82
	T1	92.1 (10.47)	88.9 (10.39)	87.8 (11.26)				
	T2	89.5 (9.39)	87.0 (10.47)	85.3 (10.73)				
**Systolic BP^b^, mean (SD)**						0.07 (2, 69)		.93
	T1	127.0 (18.36)	124.3 (19.10)	124.1 (18.77)				
	T2	123.8 (14.77)	121.3 (17.15)	120.3 (12.30)				
**Fasting glucose, mean (SD)**						3.90 (2, 69)		.03
	T1	93.9 (13.62)	92.9 (12.31)	92.2 (10.47)				
	T2	90.6 (7.90)^c^	89.2 (8.98)^c^	93.2 (10.12)^d^				
**LDL^e^ cholesterol, mean (SD)**						0.38 (2, 69)		.69
	T1	128.0 (26.67)	123.5 (35.48)	126.0 (34.77)				
	T2	129.9 (21.88)	122.5 (30.42)	123.0 (36.08)				
**HDL^f^ cholesterol, mean (SD)**						0.78 (2, 69)		.46
	T1	53.2 (14.81)	53.6 (13.20)	55.4 (14.86)				
	T2	55.0 (13.83)	57.7 (13.70)	56.9 (13.18)				
**Triglycerides, mean (SD)**						0.57 (2, 69)		.57
	T1	248.3 (372.18)	169.8 (86.41)	155.7 (104.73)				
	T2	163.1 (83.39)	130.5 (65.80)	145.0 (85.40)				

^a^WC: waist circumference.

^b^BP: blood pressure.

^c^Different superscripts indicate a statistically significant difference by Tukey least significant difference multiple comparison.

^d^Different superscripts indicate a statistically significant difference by Tukey least significant difference multiple comparison.

^e^LDL: low-density lipoprotein.

^f^HDL: high-density lipoprotein.

## Discussion

### Principal Results

We found that, compared to the control group, both the hybrid and mobile intervention groups demonstrated significantly greater improvements in heart-healthy behavioral outcomes, including composite heart-healthy behavior, its theoretical predictors (heart-healthy motivation and self-efficacy), and fasting glucose levels following the 12-week hybrid intervention for individuals at cardiovascular risk. In particular, the hybrid group—unlike the mobile group—showed significantly greater improvement in dietary behavior, a subscale of composite heart-healthy behavior, compared with the control group, and also demonstrated significantly greater improvements in interest or enjoyment, a core subscale of intrinsic motivation, than the mobile and control groups [45]. These findings suggest that the hybrid community-based heart-healthy lifestyle intervention—integrating the mobile app and motivational interviewing—demonstrated overall effectiveness comparable to the mobile app alone, while yielding greater improvements in dietary behavior and core intrinsic motivation.

The hybrid and mobile groups were more likely to increase the composite score of heart-healthy behaviors than the control group after the 12-week intervention, with no significant difference observed between the 2 groups. However, the hybrid group—unlike the mobile group—showed significantly greater improvement in dietary behavior, a subscale of composite heart-healthy behavior, compared with the control group. The finding regarding composite heart-healthy behavior suggests that motivational interviewing may not have demonstrated superiority over the use of a mobile app alone, indicating that the “My HeartHELP” mobile app itself may effectively facilitate overall improvements in heart-healthy behaviors. The “My HeartHELP” app uniquely incorporates 6 key heart-healthy behaviors simultaneously, which may significantly affect the composite score of heart-healthy behaviors. Empirically, this positive finding may be explained by the fact that the “My HeartHELP” app incorporates evidence-based behavioral change strategies [10,13,14,23], including text messaging for enhancing information, encouraging self-monitoring, and providing feedback or reinforcement in line with individuals’ behavioral goal setting.

To date, few mHealth studies on cardiovascular health have primarily relied on theory-based smartphone apps targeting multiple heart-healthy behaviors, as in our study. Most studies, by contrast, have used a variety of mHealth modalities, such as commercial apps, telephone, web-based, email, and SMS text messaging, each corresponding to a single specific behavior, such as physical activity, weight loss, or smoking [20]. Furthermore, most previous studies have predominantly emphasized health outcomes as direct indicators of effectiveness [6], without accounting for the internal validity of mHealth interventions through behavioral change mechanisms. In this regard, our study may be unique in its integration of a mobile app with evidence-based behavioral change strategies specifically designed to promote multiple heart-healthy behaviors and in its comprehensive evaluation of both behavioral changes and health outcomes, including cardiovascular parameters [14].

Meanwhile, the hybrid group, but not the mobile group, showed a significantly greater improvement in the subscale for dietary behavior after 12 weeks, compared to the control group. This finding indicates that motivational interviewing may have had an additional effect on changing dietary habits through the use of the “My HeartHELP” app. Motivational interviewing has traditionally demonstrated significant effectiveness in promoting behavioral changes in substance use, especially in diverse psychological contexts [17]. Dietary behavior involves adherence to diverse dietary guidelines and the consumption of various food groups [46], making effective intervention challenging without considering the psychological and social contexts of individuals [47]. In this regard, motivational interviewing was incorporated in the present study to mitigate the limitations of mobile apps in facilitating close expert interaction. This approach may be effective in facilitating a person-centered understanding of the psychosocial context of complex eating behavior and allows for personalized adjustments to eating behaviors, thus enhancing the intervention’s effectiveness. This highlights motivational interviewing’s particular efficacy for complex behavioral domains that require nuanced, individualized support beyond what a mobile app alone can provide.

Our findings demonstrated that both the hybrid and mobile groups exhibited greater increases in overall scores of the heart-healthy motivation compared to the control group, with no significant difference between the hybrid and mobile groups. This current study did not fully support the hypothesis that motivational interviewing would be more effective than using a mobile app alone [48,49]. Nevertheless, the hybrid group demonstrated significantly greater increases in a core subscale of intrinsic motivation—interest or enjoyment [50]—than the mobile group. In this context, the above findings may not entirely rule out the possibility that motivational interviewing could serve as an effective intervention component for strengthening intrinsic motivation [24,51], potentially offering advantages beyond the use of a mobile app alone. This finding also aligns with the conceptualization of intrinsic motivation as measured by the 4 items for interest or enjoyment in the Situational Motivation Scale [45].

However, the lack of overall statistical superiority of motivational interviewing may be explained from 2 perspectives. First, the “My HeartHELP” app incorporated substantial motivational elements, including self-monitoring, automated and tailored feedback, and goal attainment encouragement [52]. Second, the limited dosage of the motivational interviewing—4 sessions over 12 weeks—may have been insufficient to produce statistically significant additive effects across all motivational outcomes [53]. In line with this dosage, the quality and intensity of motivational interviewing delivered in this study may have constrained its effectiveness, beyond that of the mobile app alone, in fostering individuals’ autonomy to adjust behavioral goal settings, as both the mobile and hybrid groups adhered to the same predefined behavioral goals. Future research should optimize the dosage of motivational interviewing and enhance its quality of autonomy-supportive interventions, thereby more strategically and intensively enabling participants to independently design their activities and personalize goal-setting, as emphasized in self-determination theory [54].

Theoretically grounded in the IMB model [23], the observed increase in heart-healthy motivation in both the hybrid and mobile groups can be interpreted as evidence that enhanced motivation may have served as a foundational mechanism strengthening the mediating variable—heart-healthy self-efficacy—which, in turn, contributed to improvements in heart-healthy behavior (Multimedia Appendix 1). Consistent with this theoretical pathway, our path analytic data (not shown) indicated that increases in overall heart-healthy motivation scores were significantly and positively associated with increases in self-efficacy for diet (β=.32, *P*=.02) and exercise (β=.38, *P*=.005), as well as with improvements in heart-healthy behaviors (β=.27, *P*=.04). These findings may be attributable to the intervention strategies used in this study, which fostered self-directed, health-oriented internalization through self-monitoring and cognitive reappraisal—encompassing the daily input and reflection on 6 core heart-healthy behaviors via the app and motivational interviewing that encouraged participants to recognize progress and use self-management. Subsequently, self-efficacy for diet and exercise may have been enhanced through text messaging and tailored feedback reflecting individual success (eg, mastery experience) delivered via “My HeartHELP” [14], along with professional encouragement and persuasive support provided through motivational interviewing. Collectively, these cognitive and behavioral mechanisms may have contributed to the observed improvements in overall heart-healthy behaviors in either the hybrid or mobile group.

In our findings, the significantly increased self-efficacy for diet and exercise after 12 weeks of using the “My HeartHELP” app may be explained by the efficacy of the behavioral strategies embedded in the app. Goal-setting and self-monitoring can contribute to mastery experiences [55] by allowing participants to observe their own progress and achieve incremental success, reinforcing their belief in their ability to sustain heart-healthy behaviors [55]. Moreover, the “My HeartHELP” app’s personalized feedback messaging delivered daily, weekly, and monthly based on individuals’ behavioral outcomes could function as a form of verbal persuasion, as also described by Bandura [55], reinforcing self-efficacy through experts’ positive reinforcement and targeted feedback on behavioral progress. Moreover, the “My HeartHELP” app delivered 43 text messages on behavioral skills, informing users on how to apply these skills in daily life. Mobile apps are currently limited in their ability to sensitively recognize and address individuals’ emotional states. However, the 4 core principles of motivational interviewing (ie, expressing empathy, developing discrepancies, rolling with resistance, and supporting self-efficacy) are thought to be effective in addressing such emotional states [17]. Previous studies have suggested that motivational interviewing, as a behavioral intervention, can be highly effective in enhancing individuals’ self-efficacy [51]. Nevertheless, the lack of differences observed between the hybrid and mobile-only groups may be due to the strong self-efficacy effects of the mobile app overshadowing the effects of motivational interviewing.

We found no significant differences in heart-healthy information levels among the 3 groups following the 12-week intervention. All participants were provided with educational materials on cardiovascular health; the mobile group received cardiovascular information via text messaging, while the hybrid group received both text messages and individualized information. However, an analysis of the item-response rate revealed that participants in all 3 groups continued to provide incorrect responses to certain items on the Heart-Healthy Information Questionnaire at posttest—items that were nearly identical to those answered incorrectly at baseline [34]—despite completing the 12-week intervention. These findings suggest that interventionists should proactively address specific questionnaire items that participants struggled with at the baseline assessment. Integrating targeted educational reinforcement into the intervention design may be necessary to enhance knowledge acquisition and retention.

### Strengths and Limitations of This Study

This study underscored the potential of mHealth within a community setting for behavioral lifestyle interventions by optimizing user-interventionist interactions through tailored feedback delivered via in-app text messaging. To the best of our knowledge, this study is the first to reveal the effectiveness of a hybrid approach combining mobile apps and motivational interviewing counseling on heart-healthy behavioral changes, compared to mobile apps alone, within a community setting. Moreover, this indicated that the mHealth intervention using the mobile app effectively facilitated significant changes in heart-healthy behaviors, and the validity of these changes was confirmed through significant improvements in behavioral predictors based on the IMB model. This is particularly meaningful, as it establishes the internal validity of the intervention, demonstrating that the observed behavioral changes were systematically driven by theoretical constructs.

Nevertheless, this study has several limitations. First, dropouts (2 subjects in the hybrid group and 1 in the mobile group) may have led to a bias influencing the validity of the results, even though we performed an intention-to-treat analysis. Second, the absence of participant blinding in this study’s design may have introduced bias into this study’s findings. Third, because all the participants in this study were Korean, the results cannot be generalized to other ethnic groups. Fourth, because the survey was conducted online, participants with lower digital literacy were likely underrepresented. This is reflected in the socioeconomic profile of our participants, with a high proportion of college-educated individuals (64/75, 85.3%) compared with the national average in South Korea [36]. These characteristics may have facilitated greater engagement with the mHealth intervention and should be considered when generalizing the findings to populations with lower education levels or limited digital access. Fifth, this study lacks a qualitative component, which limits the understanding of why the intervention was effective from the participants’ perspective. Qualitative feedback, such as through user interviews, could have provided invaluable insight into which app features were most engaging or how the motivational interviewing sessions were perceived. Sixth, given that this study targeted multiple healthy lifestyle behaviors, appropriate behavioral and self-efficacy measures specifically designed for the comprehensive assessment of heart-healthy behaviors are lacking. Moreover, the measure of heart-healthy motivation used in this study excluded 2 subscales—relatedness and pressure or tension—because their items were deemed inappropriate for the context of adopting heart-healthy behaviors, although these dimensions, particularly relatedness, may play an essential role. The rationale for this exclusion has been described above in the Methods section. The instrument was developed and refined through a rigorous translation and back-translation process; however, the absence of a formal validation process, including factor analysis and psychometric testing, warrants cautious interpretation of the present findings. Therefore, a subsequent validation study should be conducted among the Korean population. In light of these limitations, future research should prioritize the development and validation of robust assessment tools that accurately capture the multidimensional nature of heart-healthy behavioral constructs, thereby ensuring a more precise evaluation of intervention outcomes.

### Implications for Policy and Practice

The use of a mobile app alone may be effective in facilitating changes in heart-healthy behaviors when integrated with evidence-based behavioral strategies. Given the limitations of expert-centered cardiovascular care in community settings, a mobile heart-healthy intervention led by nurses or other health professionals may serve as an efficient and accessible alternative. Although motivational interviewing did not demonstrate universal superiority over the mobile app alone, it remains a valuable component for behavioral interventions. More importantly, the hybrid approach used in this study demonstrated the potential of motivational interviewing not only to enhance intervention effects—particularly in improving intricate heart-healthy dietary behaviors—but also to promote sustainability by reinforcing intrinsic motivation, specifically interest or enjoyment [45]. We therefore strongly recommend integrating motivational interviewing into mHealth interventions, especially when targeting subtle behavioral changes and fostering core intrinsic motivation, as supported by our findings on the motivation subscales. Furthermore, the intervention could be further strengthened by integrating periodic counseling sessions using the motivational interviewing modality alongside the mobile intervention. Therefore, workforce training programs should be implemented to equip health professionals with the skills necessary to deliver mHealth interventions and motivational interviewing as components of cardiovascular health promotion. This approach could enhance the effectiveness and accessibility of heart health interventions in community settings.

### Recommendations for Further Research

Future studies should explore the long-term effects of mobile apps and their combinations with motivational interviewing on heart-healthy behavioral outcomes. Additionally, optimizing the frequency and delivery of motivational interviewing sessions should be considered, particularly within the constraints of limited resources in community settings, to enhance the sustainability and effectiveness of behavioral interventions.

### Conclusions

The hybrid community-based heart-healthy lifestyle intervention—integrating the mobile app and motivational interviewing—demonstrated comparable overall effectiveness to the mobile app alone, yet achieved greater improvements in intrinsic motivation (interest or enjoyment) and dietary behavior. These findings highlight the potential of mHealth apps as practical, stand-alone tools to promote cardiovascular health, particularly in community settings with limited access to in-person professional support. However, incorporating motivational interviewing may further enhance internalized motivation and sustain complex behavior changes over time. Health professionals can therefore adopt mHealth either independently or in combination with motivational interviewing to optimize heart-healthy behavioral outcomes. Future studies should optimize integration strategies to enhance effectiveness and evaluate the long-term sustainability of such hybrid approaches.

## Appendix

Multimedia Appendix 1 The hypothetical model based on constructs of the IMB model. IMB: Information-Motivation-Behavior. Reprinted with permission from the American Psychological Association. 2025 American Psychological Association. License obtained.

Multimedia Appendix 2 CONSORT-EHEALTH (V1.6.1) checklist.

Multimedia Appendix 3 Expanded outline of the 12-week hybrid community-based heart-healthy lifestyle intervention.

## Abbreviations

CONSORT: Consolidated Standards of Reporting Trials
IMB: Information-Motivation-Behavioral Skills
MD: mean difference
mHealth: mobile health

## Data Availability

The data supporting the findings of this study are available from the corresponding author upon reasonable request.
