# Supplementary figures and images for: Effectiveness of a Hybrid Community-Based Heart-Healthy Lifestyle Intervention: Three-Arm Randomized Controlled Trial Integrating mHealth and Motivational Interviewing
Source: JMIR Mhealth Uhealth. 2026 Jan 15;14:e76521. doi: 10.2196/76521 (PMC12856404; doi:10.2196/76521)

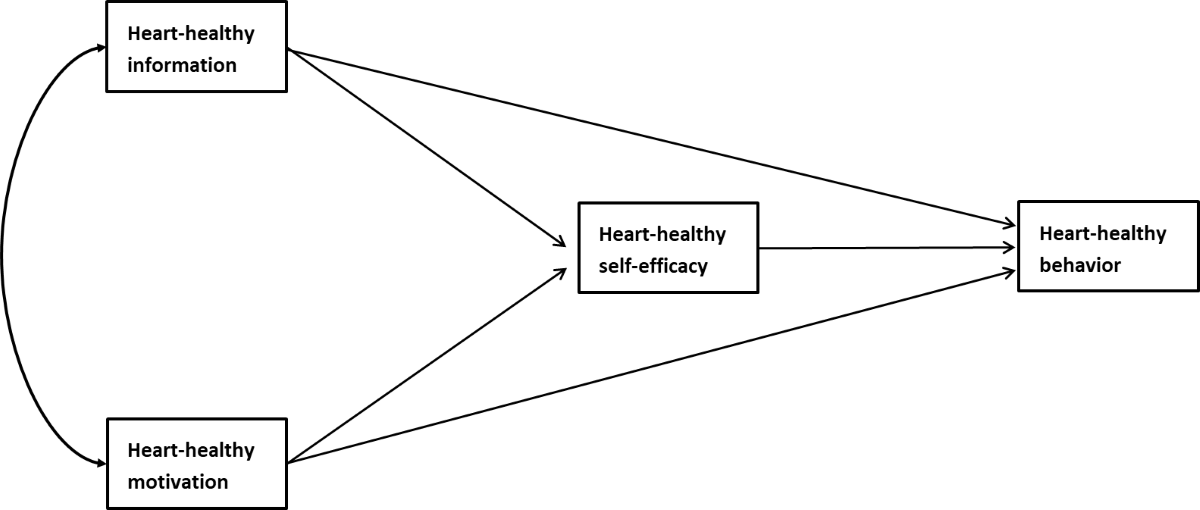

Supplement: Multimedia Appendix 1 [file mhealth_v14i1e76521_app1.png]

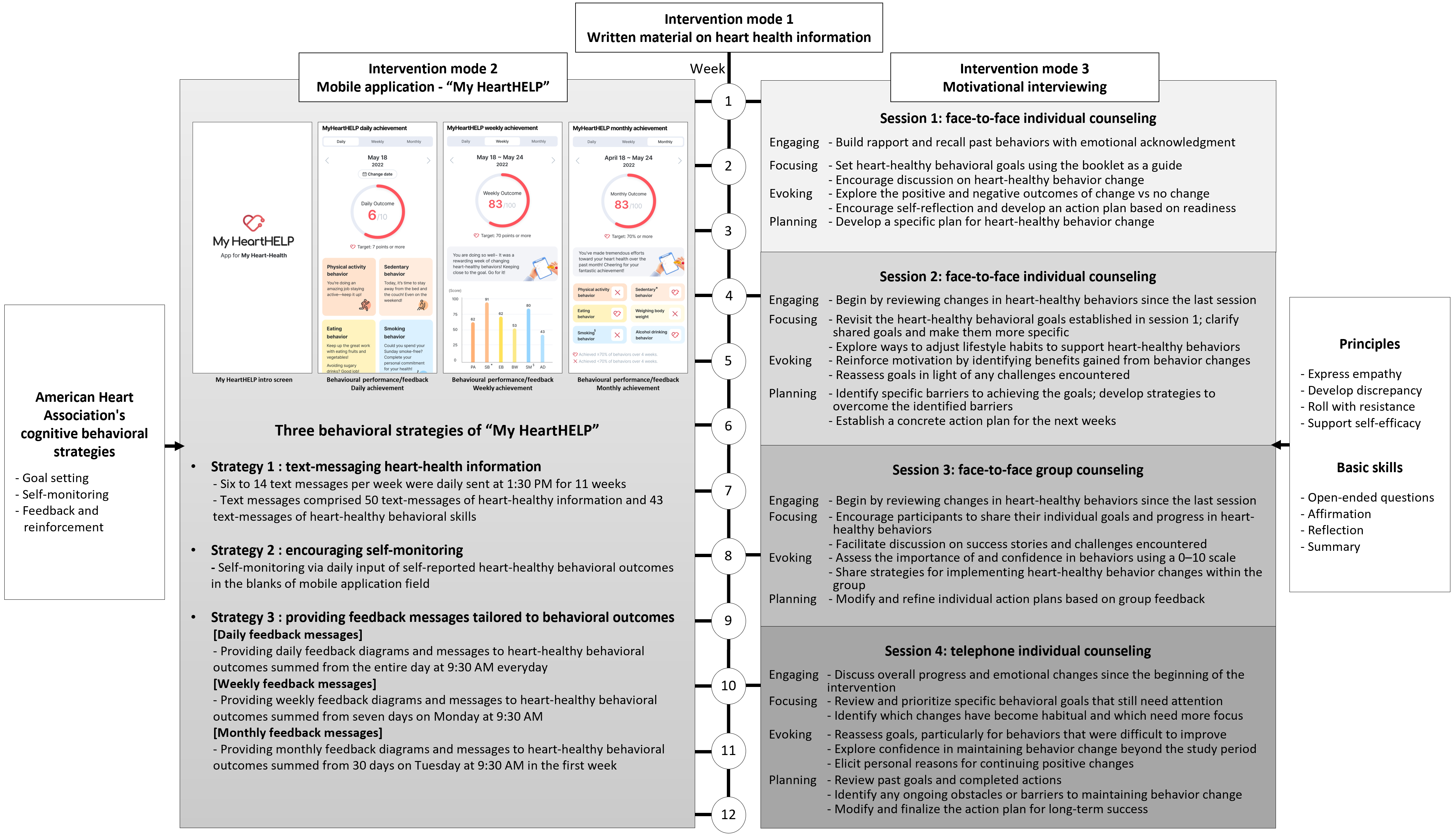

Supplement: Multimedia Appendix 3 [file mhealth_v14i1e76521_app3.png]
